# Integrated Two-Step Membrane Chromatography for Robust and High-Throughput Plasmid DNA Purification

**DOI:** 10.3390/membranes16100330

**Published:** 2026-10-02

**Authors:** Matilde Costa, Sara Cardoso, Isabela Marçal, Ângela Sousa

**Affiliations:** 1 RISE-Health, Department of Medical Sciences, Faculty of Health Sciences, University of Beira Interior, 6200-506 Covilhã, Portugal; matilde.costa@ubi.pt (M.C.);; 2 RISE-Health, Department of Chemistry, Faculty of Sciences, University of Beira Interior, 6200-506 Covilhã, Portugal; angela@fcsaude.ubi.pt; 3 Sartorius Stedim Biotech GmbH, 37079 Göttingen, Germany

**Keywords:** anion exchange chromatography, downstream processing, hydrophobic interaction chromatography, membrane chromatography, plasmid DNA

## Abstract

Plasmid DNA (pDNA) is a key raw material for advanced therapeutic modalities, including gene therapy, mRNA production, and DNA vaccination, requiring efficient and scalable downstream processing strategies. Increasing demand places stringent requirements on purification processes in terms of yield, impurity removal, preservation of plasmid topology, and robustness at scale. Membrane chromatography offers significant advantages for pDNA purification due to convective mass transfer, enabling high binding capacity at short residence times and straightforward linear scale-up. For the first time, a sequential two-step membrane chromatography platform was developed and optimized for the capture and polishing of pDNA. Capture was performed using Sartobind^®^ Q, a strong anion exchange membrane adsorber, allowing selective binding of negatively charged pDNA through electrostatic interactions with quaternary ammonium ligands under controlled buffer conditions. The capture achieved high plasmid recovery (97%) while enabling substantial removal of RNA and other process-related impurities. Process parameters were optimized to balance dynamic binding capacity, product recovery, and impurity clearance while maintaining plasmid integrity. Polishing was conducted using Sartobind^®^ Phenyl, operated under cosmotropic conditions, enabling further impurity removal and improved separation of plasmid isoforms. This step resulted in enrichment of the supercoiled (sc) pDNA fraction relative to open circular (oc) and linear forms, while maintaining high recovery (94%). The integrated process achieved an overall pDNA purity of 98%, meeting typical regulatory expectations. Process applicability was demonstrated using a second plasmid construct of larger size, highlighting the flexibility of the platform while indicating that optimization may be required for different plasmid formats. Overall, the combination of anion exchange capture and hydrophobic interaction polishing provides a robust, scale-up potential and high-throughput membrane chromatography-based platform to produce high-quality pDNA suitable for advanced therapeutic manufacturing.

## 1. Introduction

Plasmid DNA (pDNA) has emerged as a non-viral platform for gene-based therapeutics, enabling in vivo expression of therapeutic proteins, antigens, and genome editing components [1]. Its episomal nature, favorable safety profile, and compatibility with scalable manufacturing distinguish pDNA from viral vectors. Clinical validation of plasmid-based therapies, including the approval of the COVID-19 DNA vaccine ZyCoV-D, further highlights the translational potential of plasmid-mediated gene expression [2]. As clinical and commercial demand continues to grow, there is an increasing need for scalable, cost-effective, and regulatory-compliant manufacturing processes for pDNA production [2,3].

The transition from laboratory-scale preparation to industrial manufacturing requires coordinated optimization of upstream fermentation, downstream purification, and quality control strategies [3]. Advances in high-cell-density fed-batch fermentation using engineered *Escherichia coli* strains have significantly improved volumetric yields and plasmid stability [4]. However, despite these upstream improvements, downstream processing remains a critical determinant of overall process efficiency, cost, and product quality. Purification strategies must achieve efficient removal of host cell impurities while preserving the biologically relevant sc pDNA isoform, which is widely recognized as a key critical quality attribute for pDNA-based therapeutics [5].

Chromatography represents the central unit operation in pDNA downstream processing, enabling the removal of host cell RNA, proteins, endotoxins, and undesired plasmid isoforms while maintaining product integrity [6]. Conventional resin-based chromatographic systems, however, are increasingly limited by diffusional mass transfer constraints, reduced productivity, and scale-up challenges when processing large and flexible biomolecules such as pDNA. To address these limitations, convective chromatographic media, including membrane adsorbers and monolithic supports, have been increasingly adopted. These technologies enable high flow rates, short residence times, and improved binding efficiency, while supporting straightforward linear scale-up [7,8]. Such characteristics make them particularly suitable for high-throughput purification processes.

Within pDNA purification workflows, capture chromatography constitutes the primary purification step, responsible for bulk impurity removal and initial enrichment of the supercoiled (sc) pDNA fraction. Current capture strategies are predominantly based on anion exchange chromatography, leveraging the strong negative charge of pDNA to enable selective binding under controlled pH and ionic strength conditions [9,10]. Process optimization at this step focuses on maximizing binding capacity and recovery while minimizing shear-induced damage and preserving plasmid topology, in addition to ensuring robust clearance of host-derived contaminants [3,7,8].

Subsequent polishing steps are required to remove residual impurities, including trace host cell components, endotoxins, and plasmid isoform variants. Hydrophobic interaction chromatography and mixed-mode systems are commonly applied, as they provide enhanced selectivity for separating sc pDNA from oc and linear forms. This step is essential to achieve regulatory-grade purity, ensure batch-to-batch consistency, and finalize the downstream purification workflow prior to formulation [11,12,13].

Despite advances in membrane chromatography and the increasing demand for pDNA manufacturing, integrated membrane-based purification strategies combining efficient capture and polishing remain relatively underexplored. There is a need for downstream processes capable of achieving high recovery, effective impurity clearance, enrichment of the sc isoform, and high process productivity while maintaining compatibility with industrial scale-up. In this study, a two-step membrane chromatography strategy was developed for pDNA purification, integrating an anion exchange membrane-based capture step using Sartobind^®^ Q with a hydrophobic interaction membrane-based polishing step using Sartobind^®^ Phenyl. By combining two orthogonal separation mechanisms within a fully membrane-based workflow, the platform aims to reduce process complexity while maintaining high recovery and purity. The proposed approach leverages the convective mass transport properties of membrane adsorbers to support high-throughput operation and process intensification and was evaluated for its ability to efficiently remove process-related impurities and enrich the sc pDNA isoform. The results provide a framework for the development of membrane chromatography-based pDNA purification processes with potential applicability to industrial manufacturing.

## 2. Materials and Methods

### 2.1. Materials and Reagents

Chromatographic experiments were performed using Sartobind^®^ Q (1 mL and 3 mL) and Sartobind^®^ Phenyl (3 mL) membrane adsorbers (Sartorius Stedim Biotech GmbH, Göttingen, Germany). Tris base and guanidine hydrochloride (NeoBiotech, Nanterre, France), EDTA (VWR Chemicals, Radnor, PA, USA), ammonium sulfate (PanReac Applichem, Barcelona, Spain), and sodium chloride (Labchem, Zelienople, PA, USA) were of analytical grade. All solutions were prepared using ultrapure water (Milli-Q system, Millipore, Billerica, MA, USA) and filtered through 0.45 µm membrane filters (Sartorius, Göttingen, Germany) prior to use. Two plasmids of different sizes were used: pcDNA3 FLAG p53 (6.7 kbp) and HPV-16 E6/E7 (8.7 kbp), both carrying ampicillin resistance markers (Addgene, Watertown, MA, USA).

### 2.2. pDNA Production, Lysis and Clarification

For the chromatographic experiments, two plasmids (pcDNA3 FLAG p53 and HPV-16 E6/E7) were propagated in *Escherichia coli* DH5α. Cultures were grown in Terrific Broth (TB) medium containing 20 g/L tryptone, 24 g/L yeast extract, 4 mL/L glycerol, 0.017 M KH_2_PO_4_, and 0.072 M K_2_HPO_4_ (pH 7.0), supplemented with 100 µg/mL ampicillin. Cultivation was performed in Erlenmeyer flasks (25% filling volume) at 37 °C and 250 rpm overnight. Cells were harvested by centrifugation and stored at −20 °C prior to processing.

Plasmid extraction was carried out using a modified alkaline lysis protocol. Briefly, 1 g of cell pellet was resuspended in 10 mL of resuspension buffer (50 mM glucose, 25 mM Tris–HCl, 10 mM EDTA, pH 8.0), followed by addition of 10 mL lysis buffer (200 mM NaOH, 1% (*w*/*v*) SDS) and incubation at room temperature for 5 min. Neutralization was achieved by addition of 10 mL 3.0 M potassium acetate (pH 5.5), followed by incubation on ice for 20 min. The lysate was clarified by centrifugation twice at 20,000× *g* for 30 min at 4 °C. The supernatant was precipitated with 0.7 volumes of isopropanol, incubated on ice for 30 min, and centrifuged at 16,000× *g* for 30 min at 4 °C. The pDNA pellet was resuspended in 1 mL of 10 mM Tris and 10 mM EDTA buffer (pH 8.0). To further remove hydrophobic proteins and high-molecular-weight RNA, ammonium sulfate was added to a final concentration of 2.5 M. After 15 min incubation on ice, the sample was centrifuged at 16,000× *g* for 20 min at 4 °C. The supernatant was recovered and stored at −80 °C until further use in the chromatographic assays.

### 2.3. Sartobind^®^ Q Chromatography (Capture Step)

pDNA capture was performed using Sartobind^®^ Q, a strong anion exchange membrane. A 1 mL bed volume support was initially used for screening and determination of dynamic binding capacity (DBC) of pDNA using a 21.5 µg/mL sample containing 97% sc pcDNA3 FLAG p53 (6.7 kbp) and of a 19.5 µg/mL clarified lysate sample of the same plasmid containing 14% of pDNA. A 3 mL bed volume device was used for subsequent optimization of pDNA capture conditions, for robustness evaluation and integration in the sequential two-step membrane chromatography.

All experiments were conducted on an ÄKTA™ Pure system (GE Healthcare, Uppsala, Sweden) at a flow rate of 5 membrane volumes per minute (MV/min), with UV detection at 260 nm and in-line conductivity monitoring. The membrane was equilibrated with 20 mM Tris and 10 mM EDTA buffer (pH 8.0). Sample conductivity was adjusted to the target value using the equilibration buffer prior to loading. A fixed injection volume of 300 mL was applied. Elution conditions were optimized, and fractions from load, wash, and elution steps were collected for analysis. The final optimized elution gradient was performed in triplicate to assess reproducibility.

### 2.4. Sartobind^®^ Phenyl Chromatography (Polishing Step)

Polishing was performed using Sartobind^®^ Phenyl of 3 mL bed volume operated in hydrophobic interaction chromatography (HIC) mode. Experiments were carried out on an ÄKTA™ Pure system at a flow rate of 1.67 MV/min, this parameter was reduced during process development to mitigate pressure increases associated with high ammonium sulfate concentrations, with UV detection at 260 nm and conductivity monitoring. Prior to loading, samples were conditioned to the target conductivity using the equilibration buffer (3 M Ammonium Sulfate, 20 mM Tris and 10 mM EDTA buffer (pH 8.0)). A fixed injection volume of 100 mL was applied. Following loading and washing, pDNA was eluted using a decreasing ammonium sulfate gradient in 20 mM Tris and 10 mM EDTA buffer (pH 8.0). Elution conditions were optimized, and fractions were collected for analysis. The optimized method was performed in triplicate to ensure reproducibility.

### 2.5. Analytical Methods

#### 2.5.1. pDNA Quantification

Analytical chromatographic measurements were performed using an ÄKTA™ Pure system controlled by UNICORN™ 6.3 software. Separation was carried out using a CIMac™ pDNA 0.3 mL analytical column (Sartorius, Göttingen, Germany) at room temperature, with UV detection at 260 nm. External calibration curves were prepared using pcDNA3 FLAG p53 and HPV-16 E6/E7 standards in the range 1.25–50 µg/mL. The column was equilibrated with 100 mM Tris and 600 mM NaCl (pH 8.0). Samples (50 µL) were injected and separated at 1 mL/min. Elution was performed using 100 mM Tris and 1 M NaCl buffer (pH 8.0) under a step gradient (17% initial step, linear increase to 40%, followed by 100% elution buffer). Chromatograms were processed using OriginPro 2026 (OriginLab Corporation, Northampton, MA, USA). Statistical analysis was performed using two-way ANOVA with Bonferroni post hoc testing.

#### 2.5.2. Agarose Gel Electrophoresis

Agarose gel electrophoresis was performed to assess pDNA integrity and isoform distribution. A 1.0% (*w*/*v*) agarose gel prepared in Tris–acetate–EDTA (TAE) buffer (40 mM Tris, 20 mM acetic acid, 1 mM EDTA, pH 8.0) was used. Gels were stained with GreenSafe (0.012 µL/mL). Prior to analysis, samples were desalted into 20 mM Tris and 10 mM EDTA buffer (pH 8.0) using Vivaspin^®^ centrifugal concentrators (Sartorius, Göttingen, Germany). Electrophoresis was performed at 120 V for 40 min. Gels were visualized under UV illumination using a FireReader 15.15 software imaging system (UVITEC, Cambridge, UK). A 1 kb DNA ladder (GRISP, Porto, Portugal) was used for molecular-weight reference.

#### 2.5.3. Protein, Genomic DNA, and Endotoxin Quantification

Protein concentrations were determined using a BCA assay kit (Pierce, Rockford, IL, USA) following the manufacturer’s instructions, with calibration in the range 1–12 µg/µL. Endotoxin levels were measured using a chromogenic Limulus Amoebocyte Lysate (LAL) assay (ToxinSensor™, GenScript, Piscataway, NJ, USA) with a calibration range of 0.01–0.1 EU/mL. All consumables were endotoxin-free, and assays were performed under aseptic conditions. Genomic DNA (gDNA) was quantified by quantitative PCR (qPCR) using Xpert Fast SYBR Mastermix (GRISP, Portugal) on a CFX Connect™ system (Bio-Rad, Hercules, CA, USA), according to Ferreira et al. [14]. Calibration curves (0.005–50 ng/µL) were generated using gDNA standards. Primers targeting the *16S rRNA* gene were used, and amplification was performed with an initial denaturation at 95 °C for 10 min, followed by 40 cycles (95 °C, 10 s; 60 °C, 30 s; 72 °C, 15 s) and melting-curve analysis (65–95 °C). All reactions were conducted in triplicate.

## 3. Results and Discussion

### 3.1. Optimization of Plasmid Capture Chromatography Using Sartobind^®^ Q

#### 3.1.1. Dynamic Binding Capacity and Feasibility of the Capture Step

For the pDNA capture step, anion exchange membrane chromatography was implemented using Sartobind^®^ Q as the stationary phase. The interaction between pDNA and the chromatographic support and subsequent saturation is a critical performance parameter in adsorption-based chromatographic processes. This information defines the maximum amount of target molecule that can be bound under specific flow and operating conditions before significant breakthrough occurs. In pDNA chromatography, these data are typically determined by breakthrough experiments in which a pDNA-containing feed is continuously loaded under binding conditions while the effluent is monitored. High binding capacities are associated with greater loading per cycle and consequently improved process productivity, reduced membrane requirements, and lower overall purification costs [15,16].

**Figure 1:**
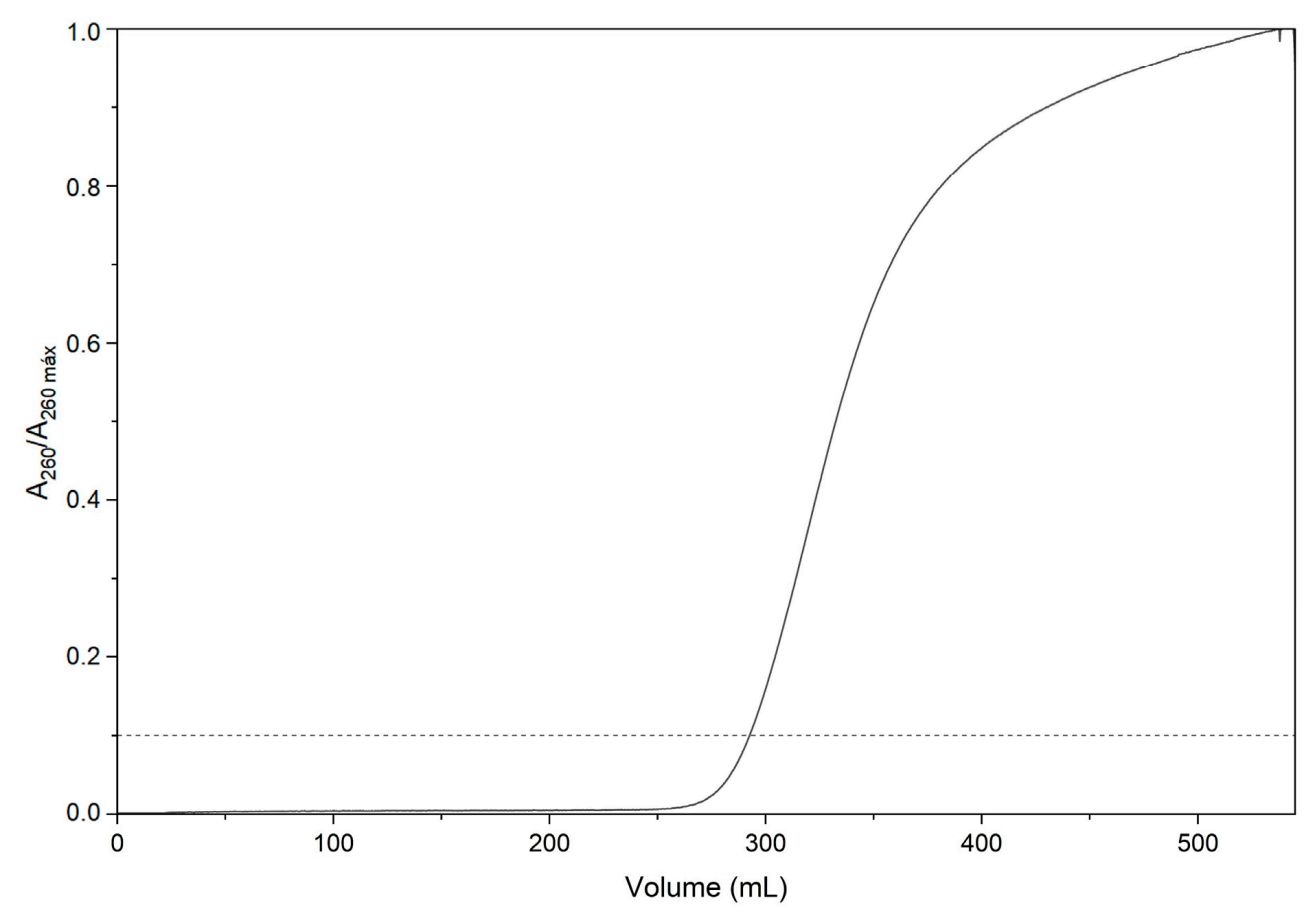
Dynamic breakthrough profile of a 97% supercoiled pcDNA3 FLAG p53 (6.7 kbp) sample loaded onto a 1 mL Sartobind^®^ Q membrane (5 MV/min) for DBC determination. Dotted line represents 10% of breakthrough.

To characterize the adsorption behavior of pDNA-Sartobind^®^ Q, a 21.5 µg/mL sample containing 97% sc pcDNA3 FLAG p53 (6.7 kbp) was loaded onto a 1 mL bed volume membrane at a flow rate of 5 MV/min, and the corresponding breakthrough curve was generated (Figure 1). Because anion exchange chromatography is generally operated under low-salt or low-conductivity conditions to promote binding of negatively charged species, the sample was diluted to support interaction of pDNA with the membrane while maintaining sufficient sample concentration. The pDNA sample was then loaded at a conductivity of 20 mS/cm, which is compatible with strong anion exchangers functionalized with quaternary amine groups and operated under low- to moderate-ionic-strength conditions [17,18]. The breakthrough curve shows a typical dynamic binding capacity profile, with an initial phase of pDNA retention followed by a sharp increase in signal until saturation is reached. Based on the sample concentration, the 10% breakthrough point corresponded to a dynamic binding capacity of 6.3 mg pDNA/mL membrane. Furthermore, saturation occurred after loading 522 mL of sample, equivalent to a total of 11.2 mg of pDNA/mL of membrane. When compared with other supports containing quaternary amine ligands, Sartobind^®^ Q shows nearly 10-fold higher capacity than beads and superporous beads. Among other membrane supports, its total capacity is within the same range; for example, Mustang Q reports 10 mg pDNA/mL of membrane and Natrix 12.4 mg pDNA/mL membrane [9].

**Figure 2:**
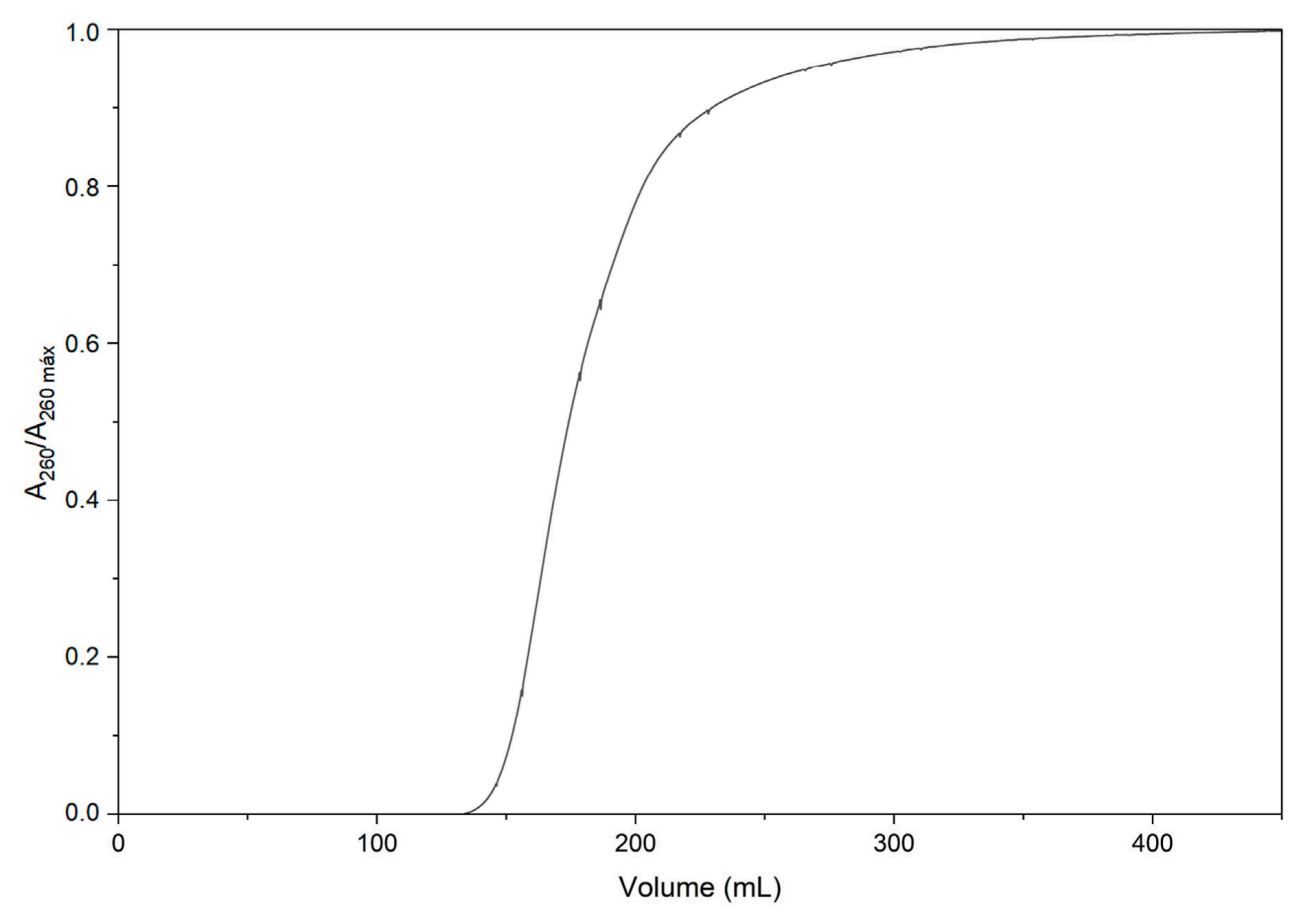
Saturation profile of a 1 mL Sartobind^®^ Q membrane (5 MV/min), loaded with a pcDNA3 FLAG p53 (6.7 kbp) clarified lysate.

A saturation curve was also generated using a pDNA clarified lysate sample (containing 14% of pDNA), loaded onto a membrane with 1 mL bed volume, at a flow rate of 5 MV/min, since this will be the starting material of the capture step (Figure 2). The sample contained 19.5 μg/mL pDNA, and the 10% breakthrough point was reached after loading 152.2 mL, corresponding to approximately 3 mg of pDNA. To define the loading amount of clarified lysate sample for subsequent optimization experiments, the loading mass was set to the corresponding 70% the initial breakthrough point (~92.5 mL), equivalent to 1.8 mg of pDNA per mL of membrane. The selected operating conditions were chosen to promote efficient binding of pDNA to the quaternary amine ligands while maintaining the high-throughput characteristics of membrane chromatography. A loading conductivity of 20 mS/cm provided sufficiently low ionic strength to favor electrostatic interactions between pDNA and the membrane, whereas a flow rate of 5 MV/min was selected to exploit the convective transport characteristics of the membrane while minimizing process time. It should also be noted that the adsorption capacity obtained with clarified lysate was lower than that observed with purified pDNA. This can be attributed not only to competition from RNA and other negatively charged impurities for available binding sites, but also to potential effects of lysate viscosity and composition on mass transfer and accessibility of ligand binding sites [19]. Consequently, the effective adsorption capacity under process-relevant conditions may be lower than the intrinsic capacity measured using purified pDNA samples.

Following DBC determination, a feasibility bind-and-elute experiment was carried out, loading 450 mL of clarified lysate containing 1.8 mg of pDNA in the membrane of 1 mL bed volume, using a linear elution gradient over 40 MV at 5 MV/min to 100% of 20 mM Tris, 10 mM EDTA, and 2 M NaCl (pH 8.0). Owing to its negatively charged phosphate backbone, pDNA exhibits a strong net negative charge under neutral to slightly alkaline conditions [20], enabling effective binding to the positively charged quaternary ammonium ligands of Sartobind^®^ Q at low ionic strength. Other negatively charged species may also interact with the membrane through electrostatic interactions but are expected to elute according to differences in charge density as ionic strength increases [18].

The initial screening results indicated that Sartobind^®^ Q has clear potential to separate RNA from pDNA during the capture step. RNA species eluted earlier in the gradient, consistent with weaker interaction with the anion exchange matrix, whereas pDNA showed stronger retention and eluted in later fractions (Figure S1). Plasmid DNA elution was completed at NaCl concentrations up to approximately 1 M, and no additional species were detected at higher conductivities. This behavior indicates that the final 2 M NaCl step was unnecessary under the tested conditions for complete pDNA elution [21]. Accordingly, the maximum NaCl concentration used in subsequent experiments was reduced, decreasing salt consumption while maintaining efficient pDNA elution and thereby improving process economy and operational robustness.

#### 3.1.2. Evaluation of Loading Sample Conductivity

Loading conductivity is a key parameter governing the interaction of lysate components with the ion exchange membrane. During sample loading, host cell proteins, low-molecular-weight RNA fragments, endotoxins, and other process-related impurities may remain unbound or interact only weakly with the membrane surface and are therefore removed during the wash step. Elution of bound pDNA is subsequently achieved by increasing salt concentration, which disrupts electrostatic interactions between the plasmid and the anion exchange ligand [22].

In this study, four loading conductivities (20, 48, 55, and 56 mS/cm) were evaluated at 5 MV/min to determine their effect on pDNA retention during capture chromatography. For these experiments, membranes with 3 mL bed volume were used instead of the 1 mL device employed for DBC determination, and the clarified lysate loading mass was proportionally increased to maintain comparable loading conditions, with same loading volume. The evaluated conductivities were selected based on preliminary screening experiments aimed at identifying the upper conductivity limit that maintained efficient pDNA binding while reducing RNA retention. In anion exchange chromatography, increasing ionic strength promotes competition between mobile-phase ions and negatively charged biomolecules for positively charged binding sites [22]. Consequently, retention of species with lower effective charge density, such as RNA fragments and other negatively charged impurities, is reduced at lower salt concentrations than that of pDNA, which possesses a high linear charge density due to its phosphate backbone. The conductivity values of 55 and 56 mS/cm were therefore selected to probe the transition region where selective impurity removal could be achieved without significant loss of pDNA binding capacity.

**Figure 3:**
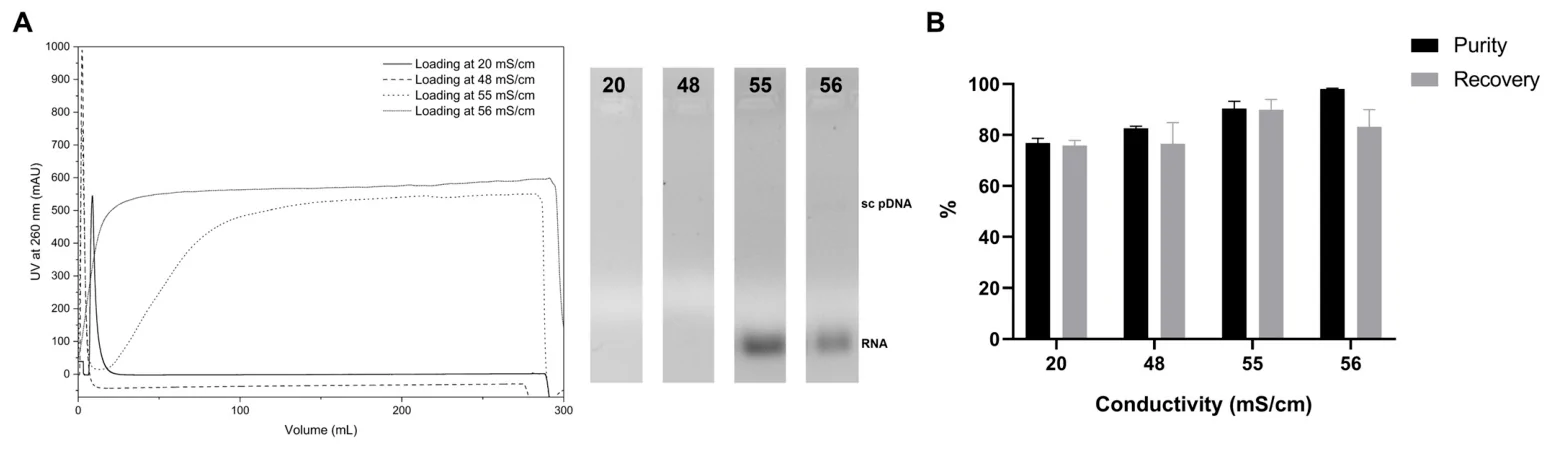
(**A**) Loading chromatograms at different conductivities (20–56 mS/cm). (**B**) Corresponding pDNA purity and recovery after capture.

As shown in Figure 3A, no major differences were observed in the UV absorbance profiles obtained at 20 and 48 mS/cm. In agreement with these observations, agarose gel electrophoresis of the corresponding loading fractions showed that nucleic acid species present in the feed were efficiently retained under both conditions. In contrast, a marked increase in UV signal during the loading phase was observed at 55 and 56 mS/cm, indicating partial breakthrough of species that were no longer fully retained at higher ionic strength. Agarose gel analysis confirmed that RNA was not retained by the membrane under these conditions. At 56 mS/cm, a higher UV absorbance was measured and a faint pDNA band became visible in the flowthrough fraction, indicating that although RNA removal was improved, pDNA retention was also compromised.

These results demonstrate that increasing feed conductivity weakens electrostatic interactions during capture chromatography and selectively affects the retention of more weakly bound species such as RNA. At the same time, excessive conductivity begins to impair pDNA binding, highlighting the need for tight control of ionic strength to ensure robust capture performance.

Bound species were eluted using a linear gradient from 0 to 100% of 1 M NaCl in 20 mM Tris and 10 mM EDTA (pH 8.0) over 80 membrane volumes at 5 MV/min. This extended gradient provided sufficient resolution to separate the different species present in the sample. Figure 3B summarizes the analytical characterization of the recovered pDNA fractions in terms of pDNA purity and recovery. Increasing loading conductivity clearly improved pDNA purity, which can be attributed to reduced RNA co-retention at higher salt concentrations during loading. However, this trend was not maintained for recovery. At the upper conductivity threshold (56 mS/cm), pDNA losses during sample loading reduced the amount of plasmid available for elution, leading to lower overall recovery.

These findings indicate that loading conductivity plays a critical role in minimizing nonspecific interactions of impurity nucleic acids while maintaining efficient pDNA binding and elution. Although two-way ANOVA with Bonferroni’s multiple comparison test did not reveal statistically significant differences among the tested conditions, a loading conductivity of 55 mS/cm was selected as the most suitable operating point, as it provided the best compromise between pDNA purity and recovery.

#### 3.1.3. Evaluation of Elution Salt

Following optimization of the binding conditions, elution parameters were investigated with the aim of increasing pDNA fraction purity. Sodium chloride (NaCl) is the conventional elution salt used in ion exchange chromatography because it disrupts electrostatic interactions in a predictable concentration-dependent manner. However, guanidine hydrochloride has increasingly been explored as an alternative because, in addition to increasing ionic strength, it may improve separation efficiency by reducing peak tailing and enhancing resolution between pDNA isoforms [23].

**Figure 4:**
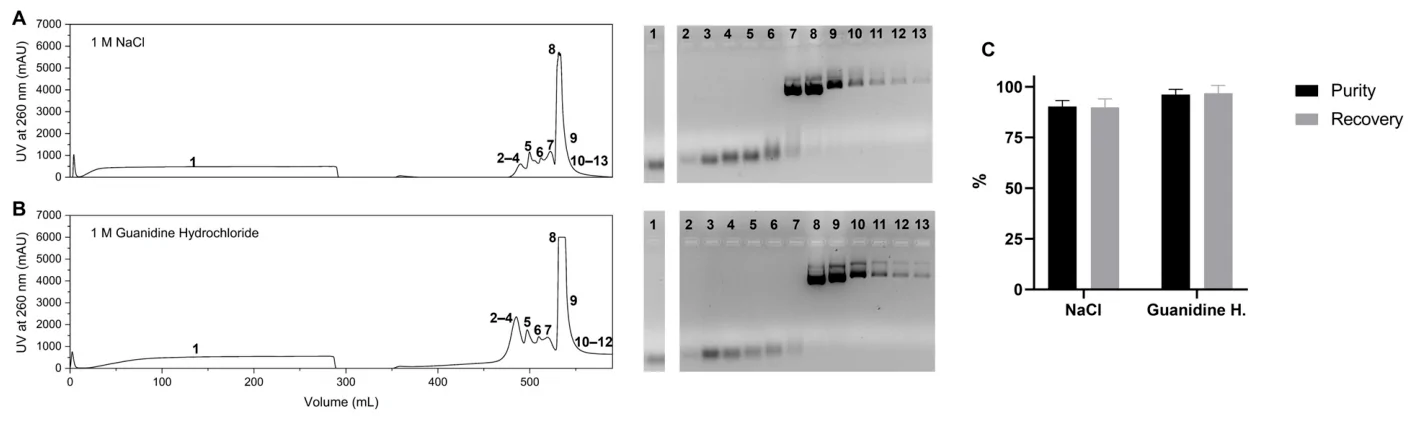
Comparison of capture elution using NaCl (**A**) and guanidine hydrochloride (**B**), with agarose gel analysis. (**C**) Resulting pDNA purity and recovery percentage.

Figure 4A,B compare the chromatographic profiles and corresponding agarose gel electrophoresis obtained using NaCl and guanidine hydrochloride as elution salts. The elution was performed in a linear gradient of 1 M NaCl/Guanidine Hydrochloride in 20 mM Tris and 10 mM EDTA (pH 8.0) over 80 membrane volumes at 5 MV/min. When NaCl was used, RNA was distributed relatively evenly across the early fractions of the linear gradient, with some co-elution with pDNA observed in later fractions. In contrast, when guanidine hydrochloride was employed, RNA eluted predominantly in the early fractions (fractions 2–4 in Figure 4B), which displayed both higher UV absorbance and more intense RNA bands in agarose gel analysis. Under these conditions, the pDNA-containing fractions showed minimal RNA co-elution. In addition, guanidine hydrochloride produced sharper elution peaks and improved baseline separation, indicating more favorable modulation of molecule–support interactions beyond simple electrostatic screening [24].

Quantitative analysis of the collected fractions (Figure 4C) showed that elution with guanidine hydrochloride resulted in modest but consistent improvements in both pDNA purity and recovery compared with NaCl. Specifically, guanidine hydrochloride yielded 96.1% pDNA purity and 96.8% recovery, whereas NaCl resulted in 90.4% pDNA purity and 89.9% recovery. These findings agree with previous reports showing that guanidine salts can improve anion exchange separation of pDNA from RNA, enabling recovery of purer product fractions without compromising yield. A plausible explanation for the enhanced resolution is that guanidinium hydrochloride, through its chaotropic effects on DNA hydration and conformation, promotes sharper separation of plasmid species than NaCl, which mainly promotes ionic interactions and therefore has a much weaker effect on resolution [23,24]. These experiments also demonstrated that, after loading approximately 300 mL of lysate, pDNA was recovered predominantly in three concentrated fractions of 10 mL each.

Although guanidine hydrochloride improved pDNA purity and recovery, its implementation at manufacturing scale requires consideration of process economics and operational aspects. Compared with NaCl, guanidine hydrochloride is generally more costly and may require additional attention regarding material compatibility and waste management. However, the improved impurity clearance achieved during capture reduced RNA carryover into the polishing step and simplified downstream processing. In the integrated process presented here, the capture eluate undergoes conditioning prior to HIC polishing irrespective of the elution salt used, partially mitigating the impact of introducing guanidine hydrochloride. Therefore, the benefits of improved purity and recovery should be balanced against the additional manufacturing considerations associated with its use. A detailed techno-economic assessment was beyond the scope of this study.

#### 3.1.4. Reproducibility Analysis

Based on the optimized conditions identified in the previous experiments, reproducibility studies were performed using a loading conductivity of 55 mS/cm and guanidine hydrochloride as the elution salt. Reproducibility was assessed by evaluating chromatographic profile consistency, including peak shape, retention time, pDNA purity, and recovery across replicate runs. All replicate experiments were performed using material derived from the same clarified lysate batch, and therefore demonstrate run-to-run reproducibility of the optimized capture process.

**Figure 5:**
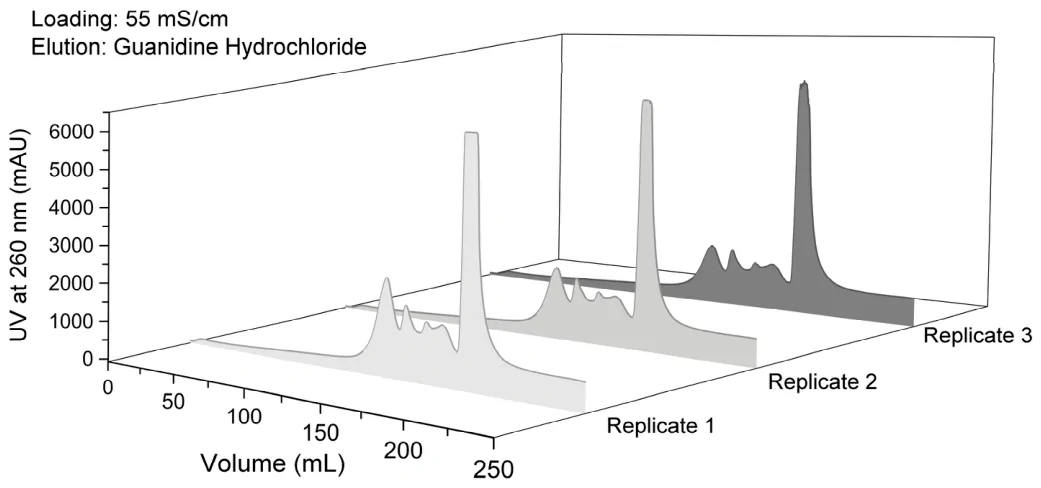
Reproducibility of Sartobind^®^ Q capture under optimized conditions (55 mS/cm, guanidine hydrochloride elution).

As shown in Figure 5, the chromatographic profiles obtained in three replicate runs using 3 mL Sartobind^®^ Q membranes were highly consistent, demonstrating reproducible pDNA binding and elution under the selected operating conditions. Table 1 summarizes the analytical results of the corresponding capture experiments performed. The similarity in peak shape and retention time across runs resulted in comparable purity and recovery values (Table 1), confirming the robustness of the optimized capture method. The data confirm that operation at elevated loading conductivity (55 mS/cm) promotes selective removal of RNA in the flow-through, while the use of guanidine hydrochloride enhances separation between pDNA and residual RNA during elution. Together, these conditions yielded highly purified plasmid fractions, with a pDNA purity of 96.1 ± 2.6% and a recovery of 96.8 ± 3.9%.

The low variability observed across replicate runs (relative standard deviation < 4%) demonstrates excellent run-to-run reproducibility and confirms the robustness of the optimized capture step.

Recent comparative studies evaluating Q-type anion exchange matrices for direct lysate purification of sc pDNA have reported similar performance for convective media. Stoops and co-workers showed that, for a plasmid with 7.3 kbp, membrane adsorbers achieved 96.1% recovery with 98% impurity reduction, while monoliths reached 98.8% recovery and 92.2% impurity reduction. Under comparable conditions, conventional resins yielded only 43.5% recovery and 74.3% impurity reduction [25]. Other reports describing quaternary amine-functionalized membranes have also shown pDNA recoveries in the range of 85–90% [26,27]. Although differences in plasmid constructs, feed composition, and operating conditions limit direct comparison, the performance obtained in the present work using Sartobind^®^ Q falls within the range reported for state-of-the-art convective purification approaches. For example, Stoops and collaborators evaluated plasmids ranging from 2.91 to 7.25 kbp using different operating conditions, including elution with 100 mM Tris and 1 M NaCl (pH 9.0) over 50 column volumes [25].

### 3.2. Optimization of Plasmid Polishing Chromatography Using Sartobind^®^ Phenyl

#### 3.2.1. Dynamic Binding Capacity of Sartobind^®^ Phenyl

For the polishing step, hydrophobic interaction chromatography (HIC) was implemented using Sartobind^®^ Phenyl. Under high-salt conditions, hydrophobic interactions between phenyl ligands and exposed hydrophobic regions of biomolecules are promoted. Separation then occurs upon decreasing ionic strength, according to differences in surface hydrophobicity and molecular conformation [28].

Breakthrough experiments were carried out using a Sartobind^®^ Phenyl 3 mL membrane, with a 96% sc pcDNA3 FLAG p53 (6.7 kbp) plasmid. The sample conductivity was adjusted accordingly, to 3 M of ammonium sulfate, and passed through a 0.45 µm filter in order to remove any precipitates. The sample at a concentration of 31.6 µg/mL, was continuously loaded at 5 mL/min (1.67 MV/min), a lower flowrate than used previously to minimize pressure increase and prevent precipitation due to high salt concentration. The experiment showed that to reach 10% saturation 4.5 mg of pDNA are needed for the 3 mL membrane, equivalent to 1.5 mg of pDNA per mL of membrane. Other chromatographic supports used for HIC, such as, monoliths functionalized with pyridine and butyl ligands present adsorption capacities of around 3 mg of pDNA/mL of support, comparable with the value obtained in this work [11,12].

In line with the strategy used for Sartobind^®^ Q, the loading amount for subsequent optimization studies was defined as 70% of the breakthrough volume corresponding to the initial detection of pDNA. Under these conditions, the selected loading corresponded to an estimated 2.6 mg of pDNA. These results indicate that Sartobind^®^ Phenyl provides sufficient binding capacity to support its use as a polishing step within the two-step purification strategy (Figure S2).

#### 3.2.2. Effect of Capture Elution Conditions on Polishing Performance

Achieving pDNA purity that meets regulatory quality criteria generally requires the integration of multiple chromatographic steps, in which the capture operation removes bulk impurities and concentrates the product, and the polishing step eliminates residual contaminants while refining plasmid isoform composition [10]. Although these operations are often optimized separately, the elution conditions used during capture can strongly affect downstream polishing performance because they directly determine the impurity profile, ionic environment, and physicochemical state of the capture eluate. Residual RNA, salt composition, and cosmotropic effects may all influence binding behavior, selectivity, and resolution during polishing chromatography [9,28].

If capture elution conditions are not well chosen, the burden on the polishing step may increase, potentially reducing separation efficiency, compromising isoform enrichment, or increasing the extent of conditioning required prior to polishing. Conversely, suitable capture conditions can simplify downstream processing by reducing impurity carryover and improving compatibility with the subsequent chromatography step.

For this reason, the impact of capture elution conditions on polishing performance was systematically investigated to understand how differences in elution chemistry affect impurity carryover, plasmid isoform behavior, and overall polishing efficiency. This interdependence is particularly important for the development of integrated, high throughput pDNA purification platforms with improved robustness and product quality.

##### NaCl-Eluted Samples

The polishing performance of capture eluates generated using NaCl was first evaluated to assess how residual impurities and elution chemistry influenced downstream HIC. As discussed in Section 3.1.3, NaCl elution from Sartobind^®^ Q resulted in partial co-elution of RNA with pDNA, producing a capture eluate with a higher impurity burden. This impurity profile was carried forward into the polishing step and directly affected chromatographic behavior on Sartobind^®^ Phenyl.

Because plasmid isoforms differ in compactness and surface exposure, hydrophobic interactions can be exploited to enrich the sc pDNA isoform while facilitating removal of residual impurities [28]. Previous studies using phenyl-based membrane chromatography have reported distinct elution behavior for pDNA isoforms, with oc pDNA eluting at approximately 190 mS/cm, sc pDNA at around 170 mS/cm, and RNA at lower conductivities [29].

**Figure 6:**
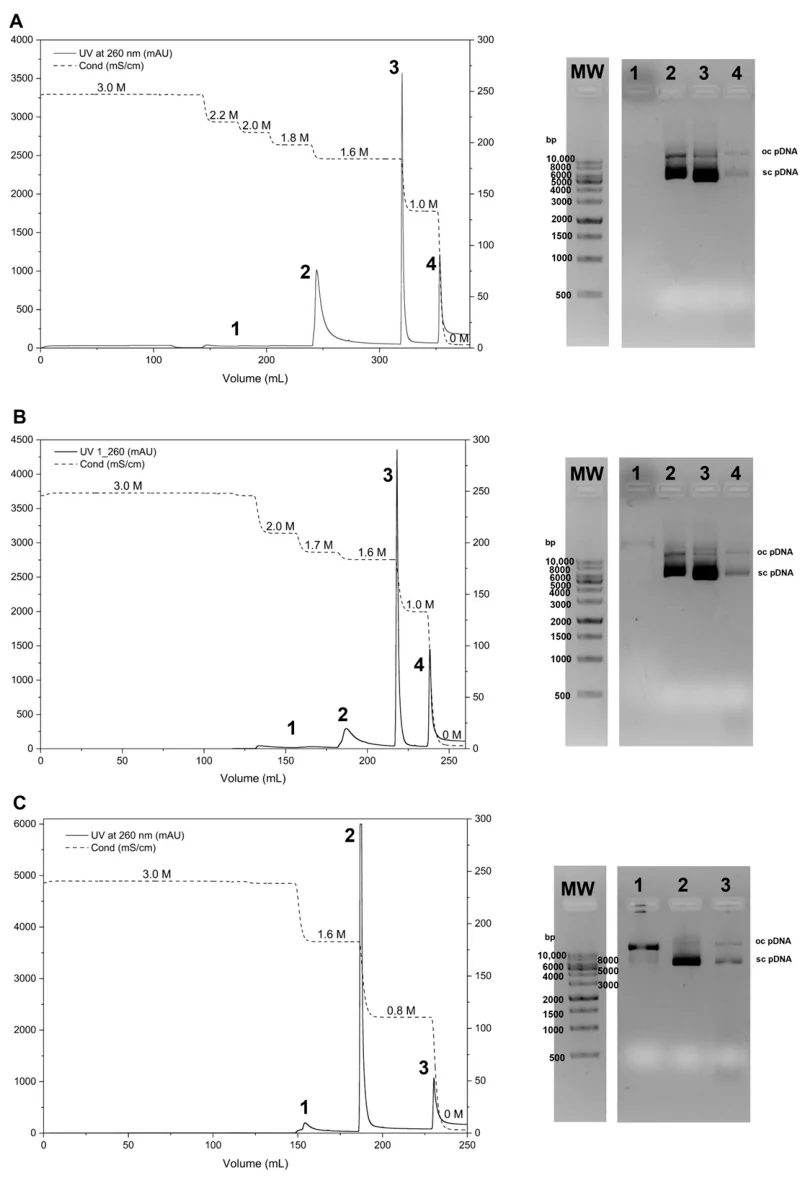
Optimization of HIC polishing on Sartobind^®^ Phenyl. Stepwise ammonium sulfate gradients showing isoform separation: (**A**) Initial screening, (**B**) intermediate, (**C**) optimized conditions.

To promote retention of all nucleic acid species under HIC conditions, Sartobind^®^ Phenyl membrane was equilibrated with 3 M ammonium sulfate in 20 mM Tris–HCl and 10 mM EDTA (pH 8.0). An initial screening experiment was then performed to characterize the elution behavior of pDNA isoforms and RNA. For this purpose, one third of the collected capture volume was injected, and both loading and elution were carried out at 5 mL/min. Small stepwise decreases in ammonium sulfate concentration were applied to achieve sufficient resolution. As shown in Figure 6A, the gradient comprised steps from 3.0 to 2.2, 2.0, 1.8, 1.6, 1.0, and 0 M ammonium sulfate, corresponding to approximate conductivities of 245, 225, 210, 200, 180, 130, and 3 mS/cm, respectively.

The results showed that oc pDNA eluted over a broad fraction range, whereas sc pDNA eluted predominantly between 1.8 and 1.0 M ammonium sulfate (approximately 190–130 mS/cm), consistent with HIC selectivity in which the more compact sc isoform exhibits higher effective hydrophobicity than oc pDNA [12].

To improve separation of the oc isoform, the elution strategy was further refined by introducing an intermediate 2 M ammonium sulfate step after equilibration at 3 M (Figure 6B). This modification enabled partial removal of oc pDNA, although some oc species were still detected in later fractions. Under these conditions, sc pDNA eluted mainly at 1.6 M ammonium sulfate, with some distribution into adjacent fractions. Analytical characterization yielded a sc pDNA purity of 95% and a recovery of 70%.

To simplify the elution profile while improving selectivity, a further optimized step-elution strategy was evaluated (Figure 6C). In this experiment, the membrane was equilibrated at 3 M ammonium sulfate and subsequently eluted at 1.65 M (approximately 180 mS/cm) and 0.8 M (approximately 110 mS/cm), corresponding preferentially to elution of oc and sc isoforms, respectively. Analytical results showed successful isolation of sc pDNA containing approximately 97.7% sc and 2.3% oc, with a recovery of 79%, representing the best overall performance achieved for isoform separation in the polishing step.

Overall, when NaCl was used as the elution salt during capture, residual RNA was still detected in the third elution step of the subsequent polishing run, indicating that trace RNA species carried over into HIC. For increased process robustness, an additional intermediate step could be introduced between the capture and the polishing steps to ensure complete RNA clearance, although this would likely cause only a minor reduction in yield (≤5%).

##### Guanidine Hydrochloride-Eluted Samples

Following optimization of polishing with NaCl-eluted material, the same step-elution strategy was initially applied to capture eluates obtained using guanidine hydrochloride. However, the resulting chromatographic profile differed from that observed with NaCl-eluted samples, and the sc pDNA isoform co-eluted with the oc fraction (Figure S3). Capture fractions obtained using guanidine hydrochloride exhibited lower RNA carryover than those eluted with NaCl, corresponding to pDNA/RNA ratios of 96.1/3.9 and 90.4/9.6, respectively. In contrast, the conductivity difference between the two eluates was minimal (approximately 2 mS/cm). These observations suggest that differences in impurity carryover, rather than conductivity, may have contributed to the altered chromatographic behavior observed during HIC, requiring adjustment of the polishing conditions. However, the underlying mechanism was not investigated in detail in this study.

**Figure 7:**
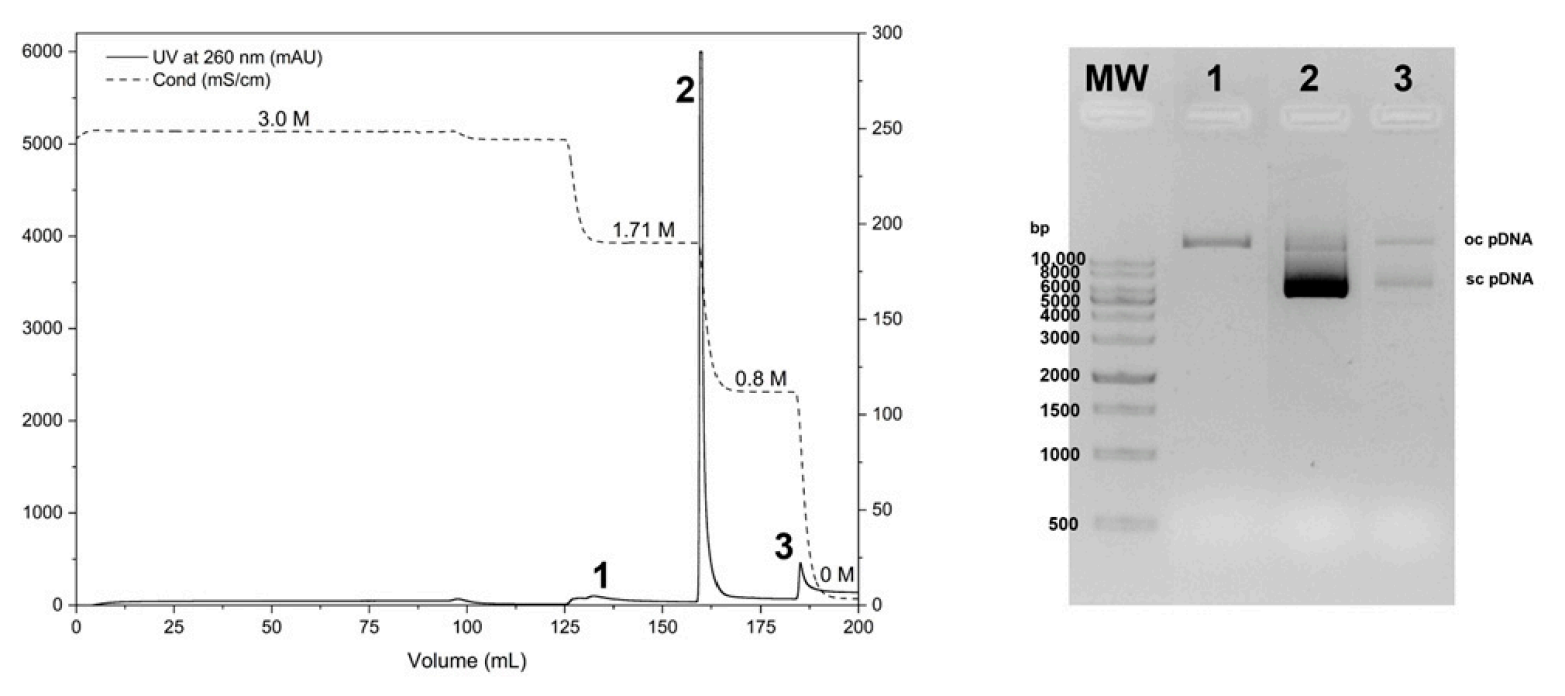
Optimized polishing profile for guanidine hydrochloride-eluted samples using stepwise ammonium sulfate gradient.

To restore effective isoform separation, the intermediate step-elution condition initially set at 1.65 M ammonium sulfate was slightly increased to 1.71 M (Figure 7). This modification improved resolution between plasmid isoforms and enabled preferential elution of the sc species while reducing co-elution of the oc form. Analytical characterization of the collected fractions showed that this optimized condition yielded sc pDNA with a sc pDNA purity of 97.4%.

These results demonstrate that polishing conditions must be fine-tuned according to the chemistry of the capture eluate and confirm that guanidine hydrochloride-eluted samples require a slightly higher ammonium sulfate concentration to achieve optimal isoform separation during HIC.

In contrast to NaCl-eluted material, capture eluates generated with guanidine hydrochloride enabled effective isolation of sc pDNA in a single elution step, with no detectable residual RNA in the downstream polishing analysis. Although the optimized method already showed high selectivity, an additional safeguard step could still be incorporated to account for trace RNA under worst-case feed conditions, again with only a marginal yield penalty (≤5%). Overall, guanidine hydrochloride provides superior robustness in terms of impurity clearance during capture, thereby simplifying downstream polishing while maintaining high purity and recovery.

##### Reproducibility Analysis

**Figure 8:**
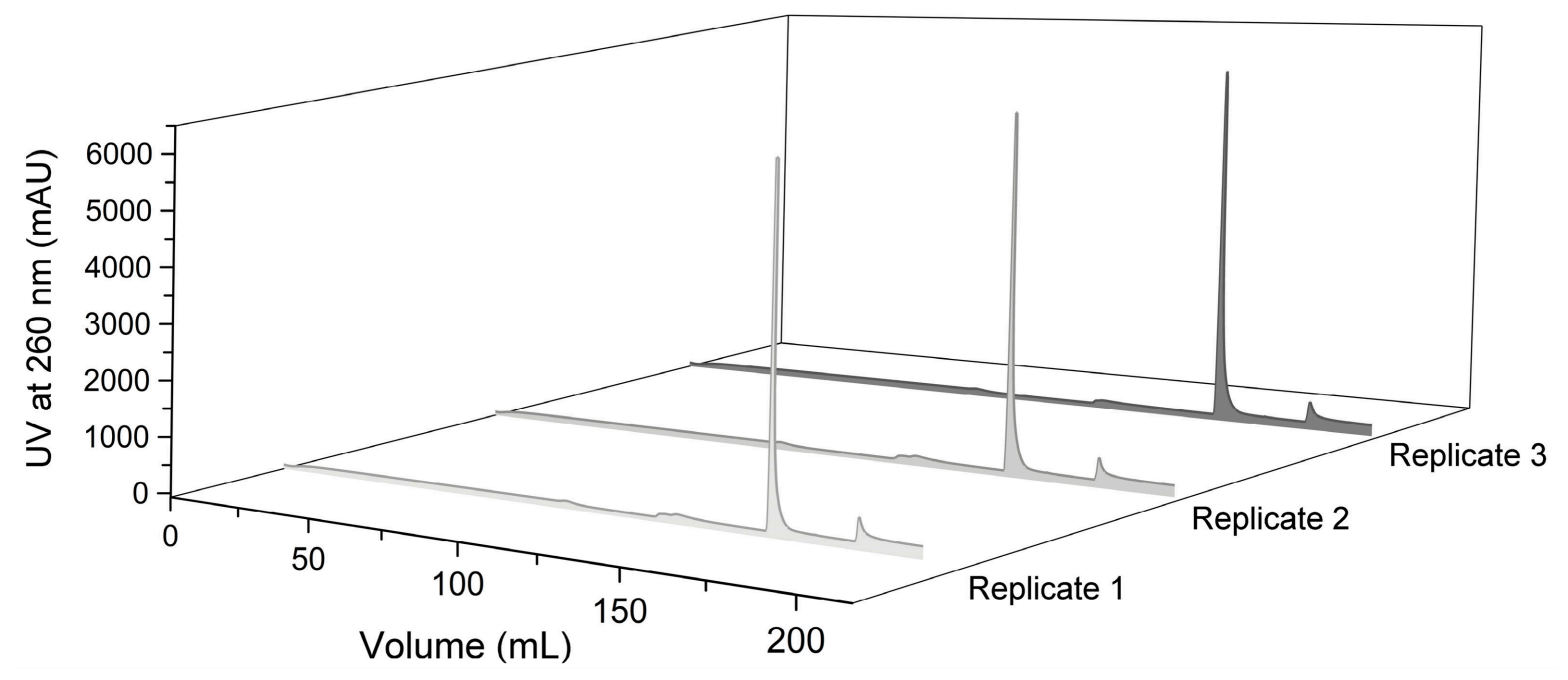
Reproducibility of Sartobind^®^ Phenyl polishing under optimized conditions.

To evaluate the robustness of the optimized polishing strategy, reproducibility studies were performed using the polishing method developed for capture fractions eluted with guanidine hydrochloride. The selected ammonium sulfate step gradient therefore consisted of 3.0, 1.71, 0.8, and 0 M ammonium sulfate (Figure 8).

The chromatographic profiles obtained in replicate runs were highly consistent, demonstrating stable binding and elution behavior under the selected conditions. The reproducibility of peak shape, elution volume, and overall chromatographic pattern confirms the robustness of the polishing step and indicates reliable performance of the Sartobind^®^ Phenyl membrane when processing guanidine hydrochloride-eluted capture material.

Analytical characterization further showed that the polishing strategy efficiently separated oc pDNA from the sc isoform, yielding a sc pDNA purity of approximately 99% (Table 2). In addition, recovery reached approximately 93%, which is in line with previously reported values for chromatographic purification of pDNA. For example, Raiado-Pereira and co-workers reported approximately 90% sc pDNA in the final fraction for a 6.7 kbp plasmid purified using phenyl-based chromatographic supports [29].

These findings are also consistent with other studies employing phenyl ligand HIC, where high purity and recovery of sc pDNA have been achieved under optimized operating conditions. Although final performance depends on variables such as support format, salt system, loading conditions, and ligand density, the purity and recovery obtained here indicate that the developed polishing step is effective for sc pDNA purification and competitive with published chromatographic methods reporting sc pDNA purities and recoveries around 96% [28,29].

### 3.3. Influence of Plasmid Construct on Two-Step Membrane Chromatography Performance

**Figure 9:**
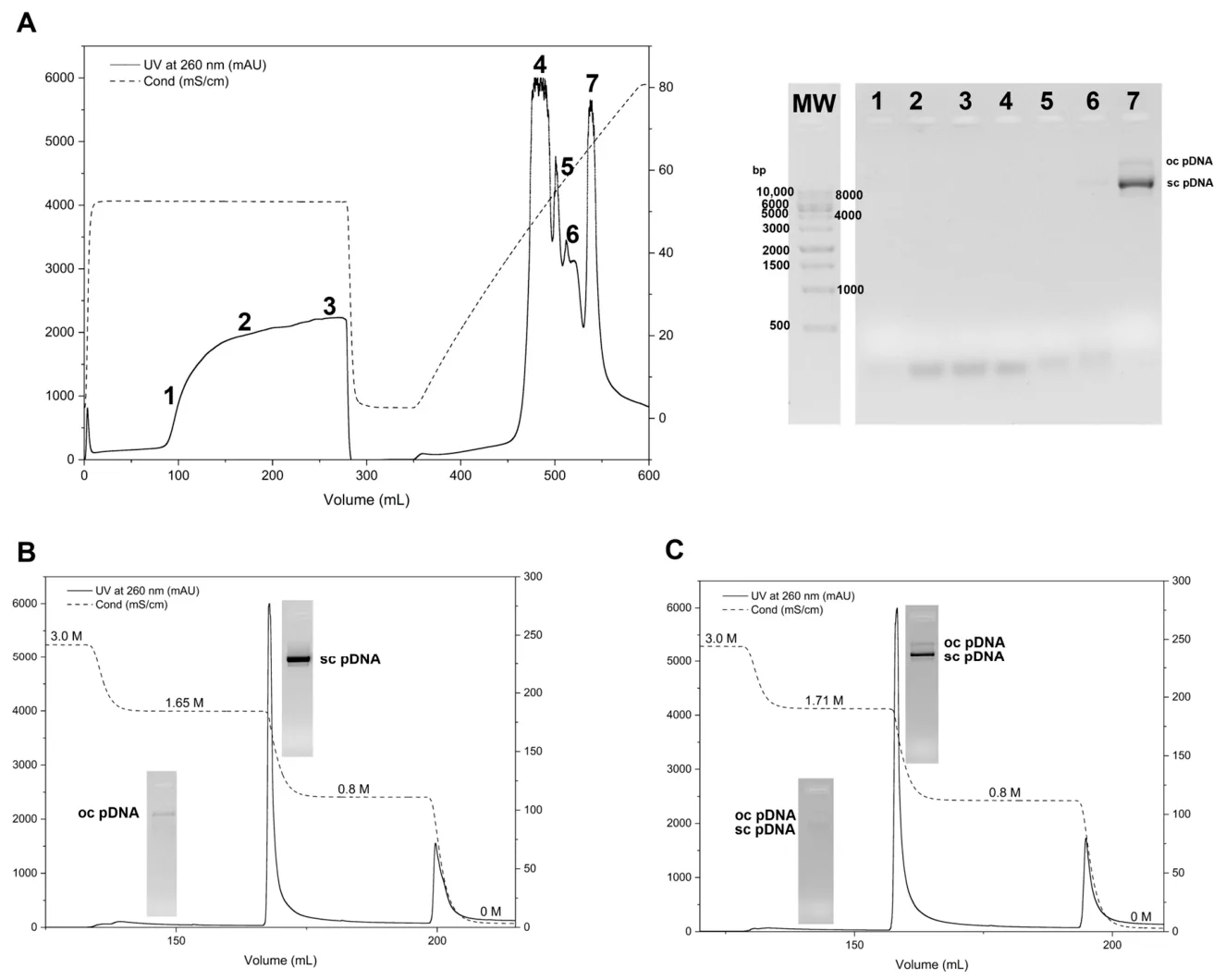
Capture and polishing of HPV E6/E7 plasmid. (**A**) Capture profile (guanidine hydrochloride elution). (**B**,**C**) Polishing under different ammonium sulfate step gradients.

To evaluate the applicability of the developed process with respect to plasmid size and feed composition, a larger plasmid, HPV E6/E7 (8.7 kbp), was investigated. For the capture step, the optimized conditions identified for the p53 plasmid were applied, namely loading of clarified lysate at approximately 55 mS/cm and elution with 1 M guanidine hydrochloride (Figure 9A).

The elevated UV absorbance observed during loading and in the early fractions of the elution gradient was attributed to the higher RNA content in the E6/E7 clarified lysate. In contrast to the pcDNA3 FLAG p53 system, where pDNA represented approximately 14% of total nucleic acids, the E6/E7 feed contained a greater relative RNA burden (around 6% of pDNA). Despite the higher RNA burden relative to the p53 feedstock, no pDNA was detected in the flowthrough, indicating that pDNA binding was not affected. However, this altered the capture elution profile, as additional RNA bound during loading and reduced peak resolution during elution. Consequently, capture performance was affected, yielding a pDNA purity of 96.8% and a recovery of 85.5%.

Because the HPV E6/E7 plasmid is larger than the construct used during process optimization and because its capture feed contained a higher RNA impurity level, its behavior during polishing could not be fully predicted. Therefore, both optimized polishing gradients were evaluated. Figure 9B,C summarize the results obtained with the two step-gradient strategies.

As shown in Figure 9B, application of the gradient consisting of 3.0, 1.65, 0.8, and 0 M ammonium sulfate resulted in clear separation between the oc and sc pDNA isoforms, although some pDNA loss was observed in the final elution step. Despite this, the sc pDNA fraction achieved a purity of 99.1% with a recovery of 92.4%. In contrast, the alternative gradient consisting of 3.0, 1.71, 0.8, and 0 M ammonium sulfate (Figure 9C) did not provide the same degree of isoform resolution and yielded a sc pDNA fraction with lower purity (92.1%).

Overall, the elution scheme comprising 3.0, 1.65, 0.8, and 0 M ammonium sulfate delivered the best performance for the larger E6/E7 plasmid and produced results comparable to those obtained for pcDNA3 FLAG p53, with differences below 1% in both purity and recovery. For comparison, Raiado-Pereira and co-workers reported approximately 90% recovery and only 60% sc pDNA purity after optimization of a 10.4 kbp plasmid purification process [29]. While the recovery values in the present study are comparable, substantially higher sc pDNA purity was achieved. This improvement may be related, at least in part, to the smaller size of the E6/E7 plasmid relative to the 10.4 kbp construct, since smaller plasmids generally exhibit lower structural heterogeneity and more favorable chromatographic behavior, facilitating improved isoform separation [29].

To confirm compliance of the purified material with regulatory quality expectations, the optimized fractions were further evaluated for gDNA, host cell protein, endotoxin content, and isoform distribution (Table 3). Overall, all plasmids showed favorable recovery yields. The p53 plasmid gave the highest recovery during capture, likely due to its lower RNA content relative to the other plasmid. During polishing, however, all plasmids showed similar recovery values, with sc pDNA percentages above 97%, meeting FDA-established criteria [30]. With respect to host–cell impurities, 99.4% of gDNA was removed overall, no protein contamination was detected in the final sc pDNA fractions, and endotoxin levels remained below the accepted threshold for the reported assay output unit [30]. The platform was successfully applied to both 6.7 and 8.7 kbp plasmids, although optimization of the polishing conditions was required for the larger construct. These results demonstrate the applicability of the process to different plasmid sizes within the range evaluated. However, larger plasmids (>10 kbp) may exhibit different chromatographic behavior due to increased size, conformational complexity, and hydrodynamic properties, particularly during hydrophobic interaction chromatography. Consequently, additional optimization of polishing conditions may be required for substantially larger constructs. Compared with conventional HIC columns, membrane adsorbers benefit from convective mass transport, short residence times, and straightforward scale-up, making them attractive for intensified processing [7]. However, differences in selectivity and plasmid-support interactions may require process-specific optimization. Further studies using a broader range of plasmid sizes would be valuable to define the operating range of the proposed platform.

Lastly, it should be noted that the clarified lysate used in this study underwent multiple pretreatment operations prior to membrane chromatography, including precipitation and centrifugation steps, which contributed to impurity reduction before capture. Consequently, the purification performance reported here should be interpreted in the context of the conditioned feedstock used for process development. While these pretreatment operations enabled systematic evaluation of the membrane chromatography platform, their impact on overall process recovery, operational complexity, and manufacturing economics was not assessed. Future studies should evaluate the integrated process using industrially relevant feed streams and include a complete mass balance across pretreatment, capture, and polishing operations.

## 4. Conclusions

This study describes the development and systematic optimization of an orthogonal two-step membrane chromatography process for pDNA purification, combining anion exchange capture on Sartobind^®^ Q with hydrophobic interaction polishing on Sartobind^®^ Phenyl. By sequentially exploiting charge-based and hydrophobic interaction mechanisms, the process enables efficient impurity removal, preservation of plasmid topology, and selective enrichment of the sc pDNA isoform.

Optimization of the capture step demonstrated that loading conductivity and elution chemistry are key parameters governing binding selectivity and impurity carryover. Operation at 55 mS/cm reduced RNA co-retention without compromising pDNA binding, while elution with guanidine hydrochloride improved separation between pDNA and residual RNA relative to NaCl. Under optimized conditions, Sartobind^®^ Q delivered high and reproducible performance, achieving pDNA purity and recovery of approximately 96% and 97%, respectively, with low run-to-run variability.

Polishing using Sartobind^®^ Phenyl further improved product quality by exploiting differences in hydrophobicity and structural compactness between plasmid isoforms. Stepwise ammonium sulfate elution enabled efficient separation of sc and oc pDNA, resulting in sc pDNA purities and recoveries up to approximately 99% and 94%, respectively. Importantly, polishing performance was strongly influenced by the impurity profile and elution chemistry of the preceding capture step, emphasizing the need for integrated optimization of sequential chromatographic operations.

Applicability of the platform was further demonstrated using a larger plasmid construct, for which the optimized capture and polishing conditions remained effective despite increased RNA burden and altered elution behavior. Although some adjustment of polishing conditions was required to maintain optimal performance. Additional studies using a broader range of plasmid sizes would be valuable to define the operating space of the process.

Overall, the results demonstrate that membrane chromatography is a robust and flexible approach for intensified pDNA purification workflows and a competitive alternative to conventional resin-based multistep processes capable of producing regulatory-grade pDNA with efficient removal of key process-related impurities, including RNA, genomic DNA, host cell proteins, and endotoxins that meet the evaluated quality criteria. Although membrane chromatography is generally considered amenable to linear scale-up through increases in membrane area while maintaining constant residence time, large-scale implementation was not experimentally evaluated in this study. The present work demonstrates process performance at the laboratory scale using commercially available membrane devices, while manufacturing-scale parameters such as pressure behavior, productivity, buffer consumption, and economic performance remain to be investigated. Future studies should evaluate these parameters at larger membrane formats to further establish the industrial applicability of the proposed platform. The high productivity, short residence times, and straightforward scale-up principles associated with membrane chromatography further support its potential for intensified manufacturing. Future work should also explore integration of the platform into intensified or continuous downstream processing workflows.

## Figures and Tables

**Table 1:** Reproducibility of capture chromatography on Sartobind^®^ Q (3 mL) under optimized conditions (purity and recovery percentages).

	Replicate 1	Replicate 2	Replicate 3	Avg ± Std
Purity (%)	98.7	96.3	93.4	96.1 ± 2.6
Recovery (%)	97.9	92.5	100.0	96.8 ± 3.9

**Table 2:** Reproducibility of polishing chromatography on Sartobind^®^ Phenyl (3 mL) (sc purity and recovery).

	Replicate 1	Replicate 2	Replicate 3	Avg ± Std
Sc Purity (%)	98.8	97.9	99.7	98.8 ± 0.9
Sc Recovery (%)	92.4	98.8	89.4	93.5 ± 4.8

**Table 3:** Summary of process performance across plasmids (pcDNA3 FLAG p53 and HPV E6/E7), including isoform distribution, and impurity clearance (gDNA, HCP, endotoxins), with quality criteria values from FDA [30]. FDA criteria reported as <1% (*w*/*w*) were converted to μg impurity/mg pDNA for direct comparison with the experimental data.

	Step	% sc pDNA	% oc pDNA	*E. coli* gDNA (µg gDNA/mg pDNA)	Host Cell Proteins (µg HCP/mg pDNA)	Endotoxins (EU/mg pDNA)
p53 (6.7 kbp)	Lysate	-	-	64.7	58.3	0.5
	Capture	96.2	3.8	15.3	Undetectable	0.1
	Polishing	97.6	2.4	0.4	Undetectable	0.1
HPV E6/E7 (8.7 kbp)	Lysate	-	-	140.2	69.5	0.4
	Capture	91.6	8.4	58.2	Undetectable	0.3
	Polishing	97.9	2.1	1.4	Undetectable	0.2
Quality Criteria	FDA	>80%	-	<10 μg gDNA/mg pDNA	<1.0 μg HCP/mg pDNA	<40

## Data Availability

The original contributions presented in this study are included in the article. Further inquiries can be directed to the corresponding author(s).

## Supplementary Materials

The following supporting information can be downloaded at: https://www.mdpi.com/article/10.3390/membranes16100330/s1. Figure S1: Feasibility run on Sartobind® Q (1 mL) using linear NaCl gradient; corresponding agarose gel analysis of fractions; Figure S2: Dynamic breakthrough profile of a 96% supercoiled p53 pDNA (6.7 kbp) sample loaded onto a 3 mL Sartobind® Phenyl membrane (5 mL/min) for DBC determination. Dotted line represents 10% of breakthrough; Figure S3: Polishing profile of guanidine hydrochloride-eluted sample using initial step-elution conditions.
