# Supplementary material for: Integrated Two-Step Membrane Chromatography for Robust and High-Throughput Plasmid DNA Purification
Source: Membranes (Basel). 2026 Oct 2;16(10):330. doi: 10.3390/membranes16100330 (PMC13706871; doi:10.3390/membranes16100330)

Supplementary Information

Figure S1 Feasibility run on Sartobind® Q (1 mL) using linear NaCl gradient; corresponding agarose gel analysis of fractions.

Figure S2 Dynamic breakthrough profile of a 96% supercoiled p53 pDNA (6.7 kbp) sample loaded onto a 3 mL Sartobind® Phenyl membrane (5 mL/min) for DBC determination. Dotted line represents 10% of breakthrough.

Figure S3 Polishing profile of guanidine hydrochloride-eluted sample using initial step-elution conditions.

Figure S1

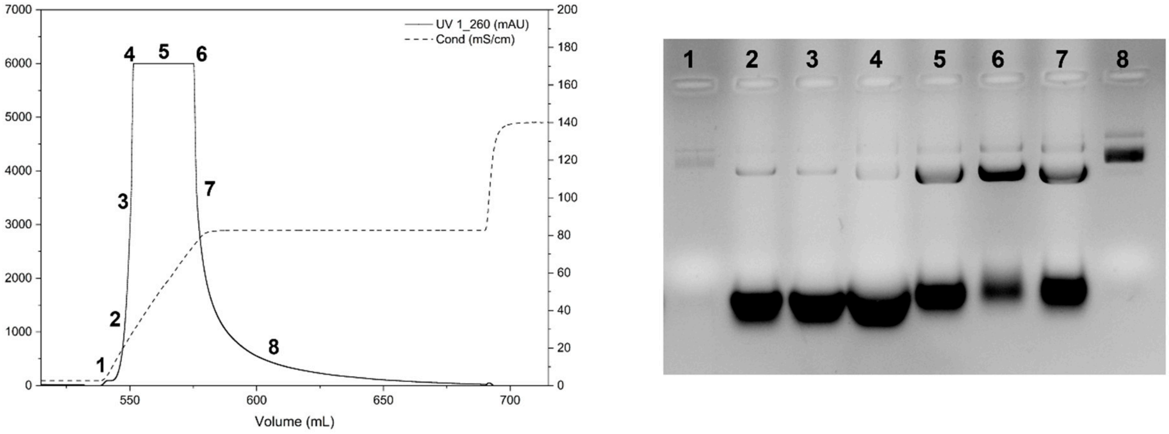

Figure S2

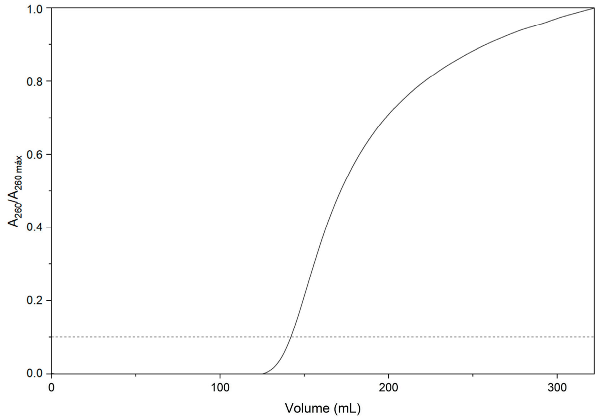

Figure S3

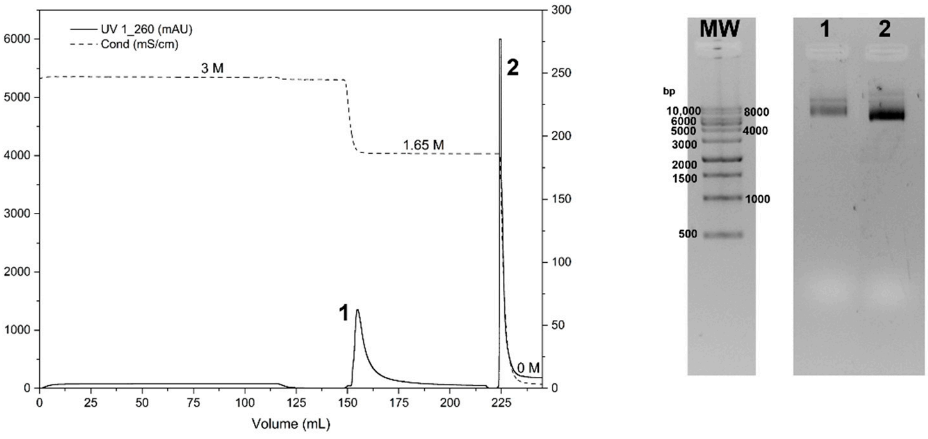

Supplement: Supplementary file 1 [file membranes-16-00330-s001.zip › membranes-4524864-supplementary.pdf]
